# Enacted and internalized stigma as predictors of successful treatment outcome among newly diagnosed tuberculosis patients in Gauteng, South Africa

**DOI:** 10.3389/fpsyg.2026.1533804

**Published:** 2026-02-24

**Authors:** T. Sole-Moloto, M. Visser, S. Mostert, V. Maduna

**Affiliations:** 1 Public Health Societies and Belonging, Human Sciences Research Council, Pretoria, Gauteng, South Africa; 2 Department of Psychology, Faculty of Humanities, University of Pretoria, Pretoria, Gauteng, South Africa; 3 Statistical Support Unit, Directorate of Research and Innovation, Tshwane University of Technology, Pretoria, Gauteng, South Africa

**Keywords:** enacted stigma, internalized stigma, predictors, South Africa, successful treatment outcomes, tuberculosis (TB)

## Abstract

**Introduction:**

Tuberculosis (TB) is a curable and preventable infectious disease that continues to pose a substantial threat to public health. A significant challenge in treating and managing TB relates to patients’ failure to adhere to TB treatment. Low adherence is often linked to stigma, which has long been recognized as one of the critical factors influencing medication adherence and significantly affects treatment outcomes. Both enacted (external stigma, experiences of unfair treatment from others) and internalized (self-stigma, beliefs in negative messages become part of how you see yourself) stigma are experienced by persons with TB (PWTB). This study examined the predictive ability of both enacted and internalized stigma on TB outcomes.

**Methods:**

A total of 90 newly diagnosed PWTB patients who were initiated on treatment were enrolled from five clinic facilities in the Ekurhuleni District, South Africa, between February 2022 and March 2023. Self-reported stigma experience was assessed using the 8-item Stigma Scale for Chronic Illnesses (SSCI-8) administered individually at enrollment among newly diagnosed participants. TB treatment outcomes were obtained after 6 months of treatment from the patients’ files, which were completed by a facility-registered nurse. Successful treatment outcomes were defined as patients who were cured or completed treatment. Bivariate logistic regression models were used to analyze the predictive ability of enacted and internalized stigma on TB outcomes.

**Results:**

The majority of PWTB (*n* = 60; 68%) experienced low stigma. A strong, significant correlation was found between enacted and internalized stigma (*r* = 0.63; *p* < 0.001). Overall, 76/87 (87%) of PWTB had favorable TB outcomes. From the bivariate logistic regression models, PWTB who had low internalized stigma and low enacted stigma were more than 2.6 times likely to have favorable TB treatment outcomes compared to PWTB with high internalized stigma and high enacted stigma. Both internalized stigma (OR = 2.6; *p* = 0.007) and enacted stigma (OR = 2.9; *p* = 0.011) odds ratios observed were significant with favorable TB outcomes.

**Conclusion:**

Although the study found low levels of internalized and enacted stigma experienced by TB patients, it remains a significant contributing factor to TB outcomes. Low stigma was associated with favorable TB outcomes among newly diagnosed TB patients. Therefore, understanding stigma in PWTB is crucial for policy development and interventions aimed at improving TB treatment outcomes.

## Introduction

Tuberculosis is an infectious disease caused by the bacterium *Mycobacterium tuberculosis*, which primarily affects the lungs (pulmonary TB) and other organs (extrapulmonary TB). Although the disease is curable and preventable, alongside HIV, TB ranks as a leading cause of death worldwide (World Health Organization, 2023). South Africa ranks 8th globally in terms of TB cases, accounting for 3.3% of the total global TB cases (Olivier and Luies, 2023). Although the country has made some progress in TB control in recent years, it still falls short of the WHO’s 90% standard for treatment success across all countries (Stop TB Partnership, 2017). A South African study, which was conducted to explore the presence of TB stigma within communities, showed that TB stigma is mainly driven and facilitated by fear of disease, coupled with an understanding of TB/HIV duality, and manifests as anticipated and internalized stigma. This means that individuals were verbally labeled with TB stigma through gossip and visually through symptomatic identification or when accessing care in either a TB-specific area in health clinics or through ward-based outreach teams (DeSanto et al., 2023). In essence, TB has increasingly become more stigmatized, especially among populations with high rates of TB and HIV co-infections (Craig et al., 2017) because of its biological, epidemiological, and social interactions with HIV/AIDS (Bresenham et al., 2020). Tuberculosis-related stigma continues to pose a formidable challenge for TB prevention and control (Datiko et al., 2020) and has been identified as an obstacle to patients seeking medical care and completing a full course of treatment (Chen et al., 2021). Patients may not take their medication as prescribed or avoid disclosing their TB status for fear of being judged, isolated, and rejected by others. Therefore, higher stigma negatively affects adherence (Pradhan et al., 2022). Both enacted and internalized stigma are experienced by people diagnosed with TB. Internalizing stigma may increase the risk of mental and physical health problems, which can have negative consequences for treatment adherence (Blake Helms et al., 2017). Feldhaus et al. (2018) found that self-stigmatization has a significant influence on patients’ medication adherence attitudes. Furthermore, internalized stigma (self-stigma) is linked to increases in psychological distress and poorer quality of life (Cheng et al., 2019; Sarkar et al., 2019). TB patients’ behavior in response to social discrimination can contribute to diagnostic delays, non-adherence, and the abandonment of treatment, which can result in an increased number of multidrug-resistant tuberculosis cases (Marahatta et al., 2020; de Almeida Crispim et al., 2017). The impact of TB stigma remains underexplored and underappreciated (Bresenham et al., 2020), and more research, using validated instruments to quantify the impact of stigma on treatment compliance, is needed (Maleche et al., 2017). This study aimed to examine the predictive ability of enacted and internalized stigma for TB outcomes using a quantitative research approach.

## Materials and methods

### Study design, context, and participants

This is an observational (i.e., cohort) study without randomization or any control. The study was conducted in Ekurhuleni Metropolitan Municipality, Gauteng Province, South Africa. The City of Ekurhuleni is one of the most densely populated areas in the country, and according to the South African National Strategic Plan for HIV, TB, and STIs 2017–2022, it is one of the districts with very high HIV and TB burdens. The study was conducted with PWTB between February 2022 and March 2023 at five clinic facilities around the Ekurhuleni District. The selection of these facilities as the research setting was based on the convenience, availability of TB initiation and patient follow-up services, research infrastructure for TB burden, free treatment services, and the risk of non-adherence/negative treatment outcomes. Therefore, TB patients who met the following inclusion criteria were recruited: (1) aged 18 years or above; (2) newly diagnosed drug-sensitive (DS) TB patients (diagnosed at the clinics); (3) diagnosis of DS-TB (e.g., pulmonary TB): TB was diagnosed and the facility-registered nurse ruled out MDR/XDR TB; (4) newly diagnosed DS-TB patients who were initiated on standard TB treatment at the clinics (based on Xpert ^®^MTB/RIF results and rifampicin resistance pattern for current TB episode, smear test results, culture test results, chest X-ray results, if available); and (5) English proficiency: willing to be interviewed in English and in-person (multiple languages were also used to explain complex concepts). Adult patients dually infected with TB and HIV were excluded from the sample because the investigators wanted to study TB stigma and not stigma associated with HIV.

### Data collection and procedure

The facility-registered nurse assisted the study team with initial patient recruitment at the clinic until the target sample size was reached. Before recruitment, the facility-registered nurse was briefed by the researcher on the exclusion/inclusion criteria for patient recruitment. Data collection began once a newly diagnosed TB patient who met the inclusion criteria expressed interest in the study. Following the registered nurse’s referral, the study team met with the potential participants and conducted quality checks to ensure the referred patients met the inclusion criteria. They were provided with study information and informed consent documentation. Patients were told that they could withdraw from the study without any negative consequences to their TB treatment. Participants who agreed to participate completed a written informed consent document before data collection commenced. Data collection was conducted in the clinics’ consultation rooms. The study used identity barcodes to link patients to their facility files and to obtain TB outcomes (to which the facility nurse granted the research team access) after 6 months of treatment.

The investigator and research assistants assisted 90 patients in completing the stigma scale individually. To ensure data quality, the investigator trained the research assistants and piloted the instrument to identify areas needing attention. The collected data included sociodemographic and stigma information, as well as overall TB outcomes.

A structured biographical questionnaire was used to collect sociodemographic information (gender, age, household characteristics, education level, and employment status), the date of diagnosis, and the start of treatment. The study team administered the questionnaire, and patients completed it during the first consultation.

The Stigma Scale for Chronic Illnesses 8-item version (Molina et al., 2013) is designed to measure internalized (three items) and enacted (five items) stigma experienced by people with neurological conditions. An example of an item for internalized stigma is: “I felt embarrassed about my illness”; whereas for enacted stigma an item reads: “Some people acted as though it was my fault I have this illness”. The researcher used the SSCI-8 scale for TB because it is classified as a chronic disease. Each item on the scale is rated on a 5-point Likert scale ranging from 1 (never) to 5 (always). The study used direct sum scores which ranged from 8 to 40, with higher scores indicating higher levels of perceived stigma. The SSCI-8 has shown high internal consistency (Cronbach’s alpha of 0.89) (Molina et al., 2013), although the scale has not been used in South Africa before, it has been used in TB populations, one in Kenya (Marangu et al., 2017), and recently in India (Elavarasi et al., 2024). Study data were collected and managed using REDCap electronic data capture tools hosted at the South African Medical Research Council (SAMRC) (Harris et al., 2019; Harris et al., 2009). REDCap (Research Electronic Data Capture) is a secure, web-based software platform designed to support data capture for research studies, providing (1) an intuitive interface for validated data capture; (2) audit trails for tracking data manipulation and export procedures; (3) automated export procedures for seamless data downloads to standard statistical packages; and (4) procedures for data integration and interoperability with external sources.

The TB outcomes were interpreted as successful (when a patient has completed a TB treatment regimen and their outcome is indicated as cured or completed) or unsuccessful (when the patient’s treatment outcome is shown as loss to follow-up or treatment failure). Interpretation of these outcomes was adopted from the National Tuberculosis Management Guidelines (National Department of Health, 2014), as shown in Table 1. Treatment outcome was determined by the facility nurse after 6 months of treatment and documented in the facility’s files. The research team obtained participants’ TB outcomes from their files.

**Table 1:** Interpretation of overall treatment outcomes.

Outcome	Definition
Cure	Patient whose baseline smear (or culture) was positive at the beginning of the treatment and is smear/culture-negative in the past month of treatment and on at least one previous occasion at least 30 days prior.
Treatment completed^*^	Patient whose baseline smear (or culture) was positive at the beginning and has completed treatment but does not have a negative smear/ culture in the past month of treatment and on at least one previous occasion more than 30 days prior. The smear examination may not have been done or the results may not be available at the end of treatment.
Treatment failure	Patient whose baseline smear (or culture) was positive and remains or becomes positive again at five months or later during treatment. This definition excludes those patients who are diagnosed with RR-TB or MDR-TB during treatment.
Died	Patient who dies for any reason during the course of TB treatment.
Treatment default (loss to follow-up)	Patient whose treatment was interrupted for two consecutive months or more during the treatment period.
Transfer out	Patient who was referred to a facility in another district to continue treatment and for whom the treatment outcome is not known.

^*^Treatment success indicated by a combination of the patients who were cured and those who completed treatment.

Source: South African National Department of Health (2014).

### Statistical analysis

All data analyses were performed using IBM SPSS Statistics version 29. Medians and interquartile ranges for continuous data, and the frequencies and percentages for categorical data (i.e., sociodemographic information and TB outcome data) were analyzed. Spearman’s correlation analysis was used to assess correlations between stigma and overall TB treatment outcome. Bivariate logistic regression analysis was used to analyze the predictive ability of stigma on overall TB treatment outcome. All comparisons were two-tailed, and *p* < 0.05 was considered statistically significant.

## Results

### Demographic and socioeconomic characteristics

**Figure 1:**
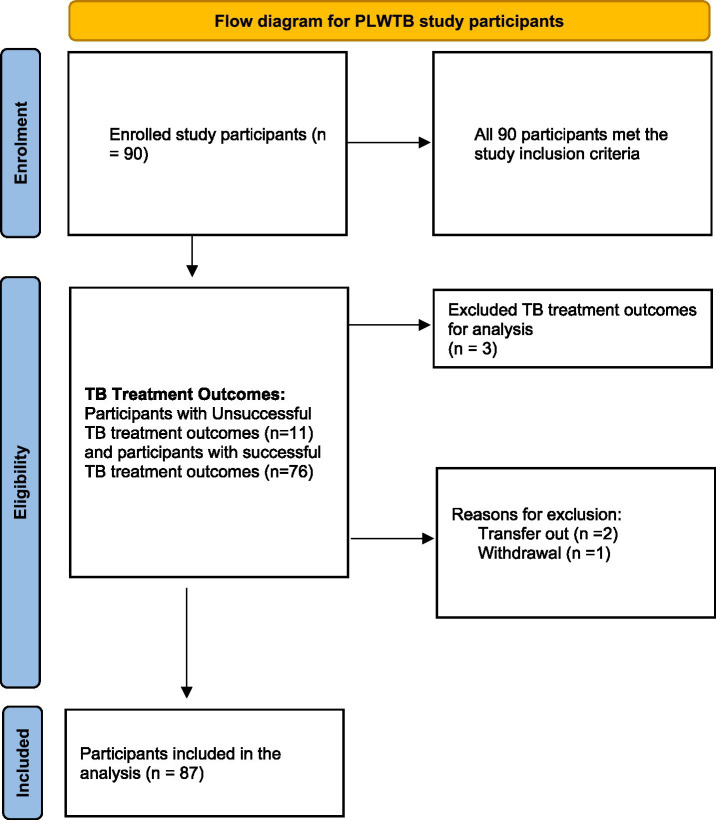
Study participants’ flow diagram.

The sample included more males (*n* = 64: 73.6%) than females (*n* = 23: 26.4%). The age range of the patients was 21 to 82, with a mean age of 40. The majority of patients were single (*n* = 59; 68%) and the socioeconomic status indicated a high unemployment rate of 58.1% (*n* = 50). Approximately 40% of patients had completed matric/Grade 12. Of the 90 patient records, 87 records had treatment outcomes documented. About 87.44% (*n* = 76) of patients had successfully completed TB treatment and their outcome results were indicated as cured (*n* = 22) or completed (*n* = 54). Unsuccessful TB treatment outcomes accounted for 12.6% (*n* = 11)—see Table 2. In the study context, unsuccessful treatment outcomes were characterized by a combination of lost to follow-up (*n* = 3), or died (*n* = 3), or treatment failure (*n* = 2), or retreatment (*n* = 1) and resistant to treatment (*n* = 2). It is important to note that 90 patients completed the stigma scale but only 87 were left after withdrawing transfer out and withdrawal categories to determine adherence. Figure 1 presents the study participants in a flow diagram.

**Table 2:** Demographic and socioeconomic characteristics of the sample.

Variables	*N* (%)	Overall stigma status^*^	
		Low stigma	High stigma
		(*n* = 35)	(*n* = 52)
Gender, *n* (%)			
Male	64 (73.6%)	22 (62.9%)	42 (80.8%)
Female	23 (26.4%)	13(37.1%)	10 (19.2%)
Age			
Age range, min and max	21 and 82	22–82	21–73
Mean (years)	40	39.6	40.8
Marital status, *n* (%)			
Married	14 (16.1%)	6(22.9%)	6(11.5%)
Single	59 (67.8%)	18(51.4%)	41(78.9%)
Living with partner	8 (9.2%)	7(20.0%)	1(1.9%)
Separated	1 (1.2%)	1(2.9%)	0(0.0%)
Divorced	1 (1.2%)	0(0.0%)	1(1.9%)
Widowed	4 (4.6%)	1(2.9%)	3(5.8%)
Residential conditions, *n* (%)			
Brick house on separate stand	62 (71.3%)	24(68.6%)	38(73.0%)
Shack	18 (20.7%)	7(20.0%)	11(21.2%)
Hostel	5 (5.8%)	2 (5.7%)	3 (5.8%)
Flat	2 (2.3%)	2(5.7%)	0(0.0%)
Employment status, *n* (%)			
Full-time	14 (11.3%)	7(20.6%)	7(13.5%)
Part-time	10 (11.6%)	3(8.8%)	7(13.5%)
Self-employed	6 (7.0%)	4(11.8%)	2(3.9%)
Unemployed	50 (58.1%)	16(47.1%)	34(65.4%)
Retired	6 (7.0%)	4(11.8%)	2(3.9%)
Highest level of education, *n* (%)			
No schooling	8 (9.2%)	0(0.0%)	8(15.4%)
Some schooling	2 (2.3%)	1(2.9%)	1(1.9%)
Completed primary school	5 (5.8%)	4(11.4%)	1(1.9%)
Completed secondary school	3 (3.5%)	2(5.7%)	1(1.9%)
Partially completed high school	30 (34.5%)	8(22.9%)	22(42.3%)
Matric/Grade 12	35 (40.2%)	18(51.4%)	17(32.7%)
Certificate/Diploma	3 (3.5%)	2(5.7%)	1(1.9%)
Bachelor’s degree	1 (1.2%)	0(0.0%)	1(1.9%)
Overall treatment outcome, *n* (%)			
Successful	76 (87.4%)	32(91.4%)	44(84.6%)
Unsuccessful	11 (12.6%)	3(8.6%)	8(15.4%)

^*^The scale ranges include low, medium, and high stigma. For the analysis, interest was on low (any score below the total) versus high (scoring a total).

### Stigma scale scores

The Cronbach’s alpha for stigma was above 0.80 (refer to Table 3) and well above the ideal benchmark of 0.7 (DeVellis and Thorpe, 2021). Therefore, the items in the scale can be regarded as sufficiently consistent and indicate that the measure is reliable.

**Table 3:** Summary of reliability analysis of the stigma scale instrument.

Scales	Number of items	Average inter-item correlation	Cronbach’s alpha score
Internal stigma	5	0.4904	0.8279
Enacted stigma	3	0.5726	0.8008

**Figure 2:**
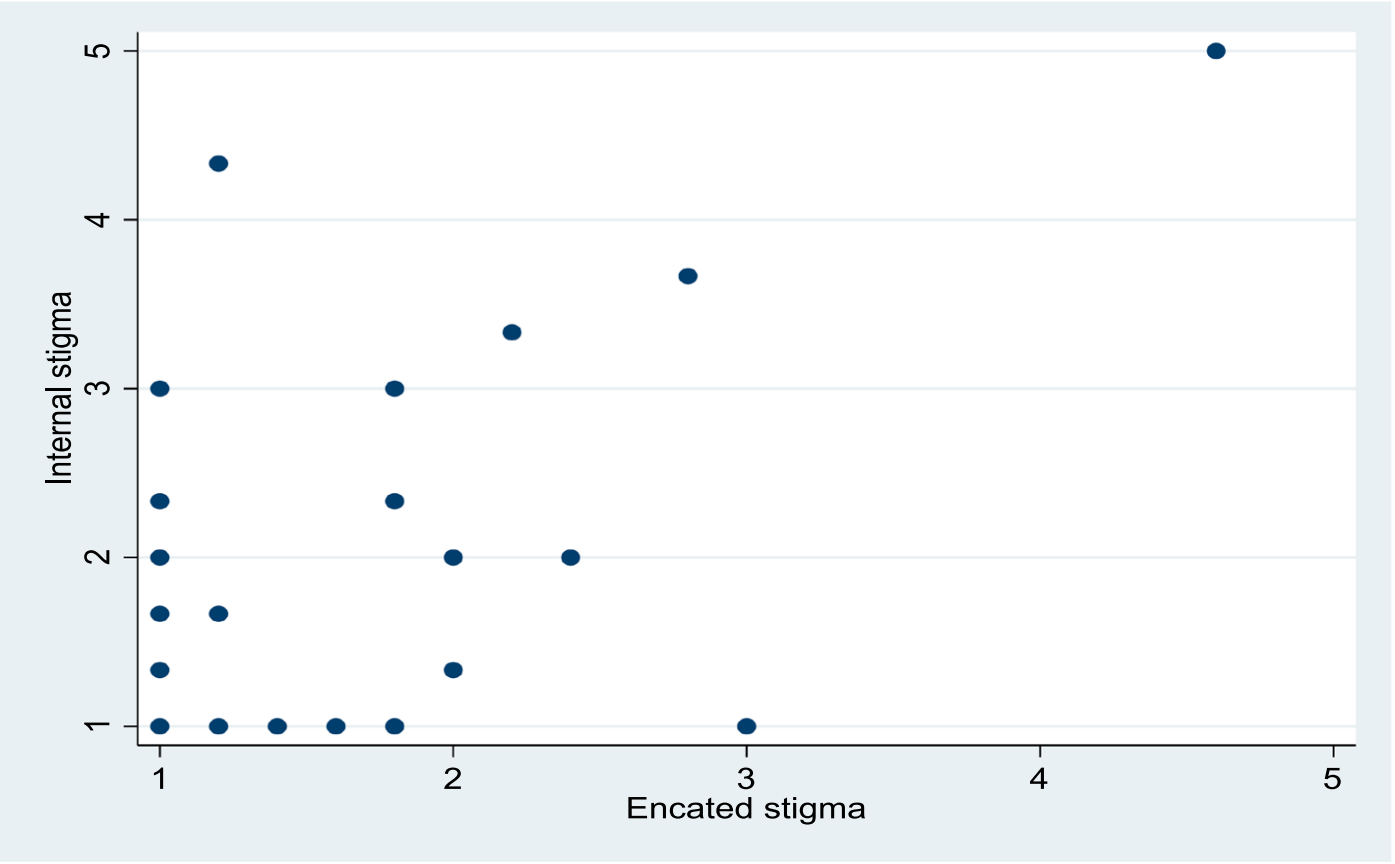
Scatter plot between internal stigma and enacted stigma.

The majority of PWTB (*n* = 60; 68%) experienced low stigma on both stigma scales (i.e., range of 8 to 10) on a scale of scores ranging from 8 to 40. The Spearman’s rho showed a strong positive significant correlation between enacted and internalized stigma (*r* = 0.63; *p* < 0.001), with high levels of enacted stigma associated with high levels of internalized stigma. The effect explains 39.69% of the total variation. Figure 2 illustrates this relationship using a scatterplot.

**Figure 3:**
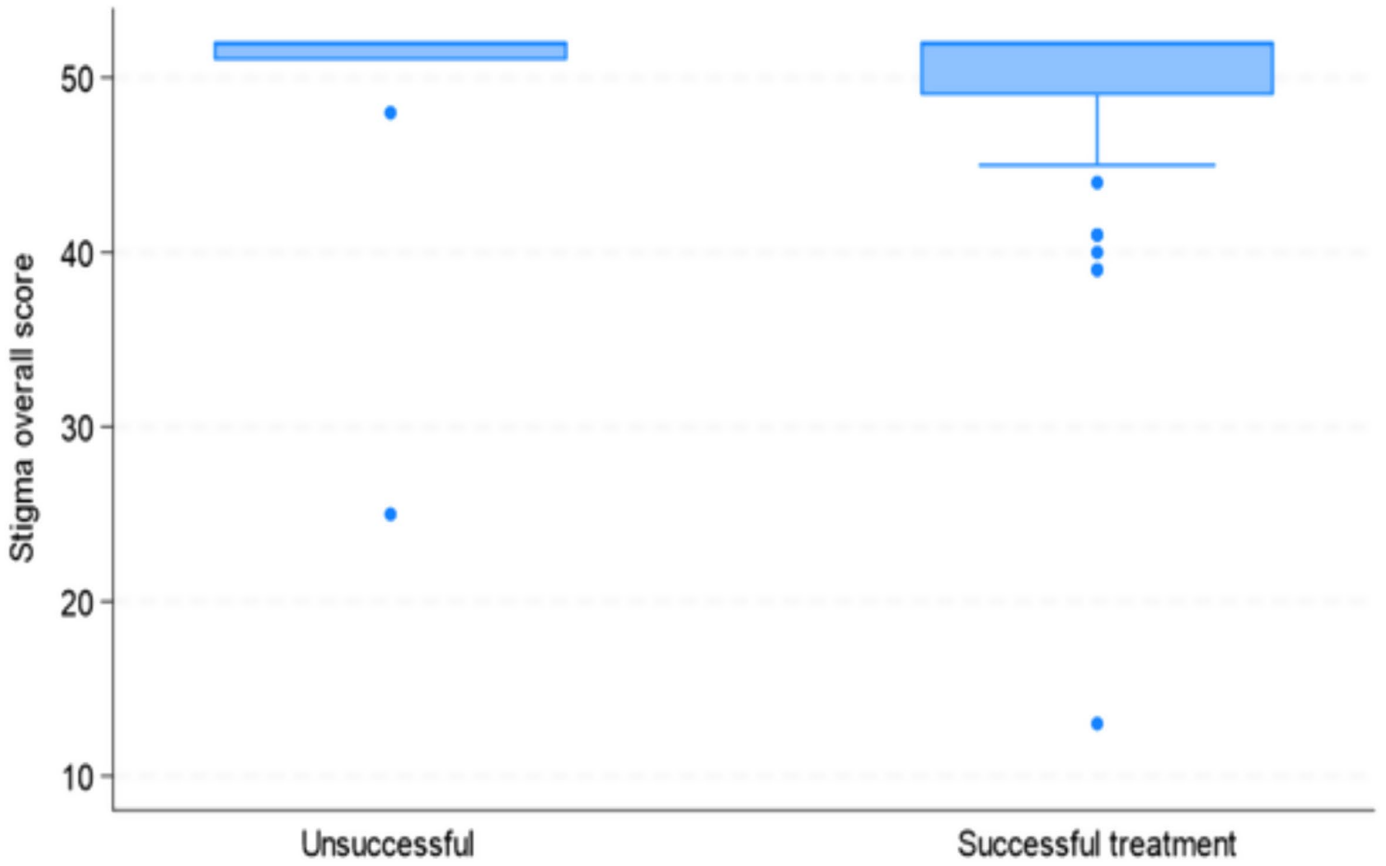
Boxplot between stigma and TB treatment outcome groups.

The rank biserial correlation was also used to assess the association between stigma and the successful and unsuccessful treatment groups. Although the unsuccessful treatment group showed slightly higher median stigma scores, the effect size was negligible (r = 0.094), suggesting that the association between stigma and treatment outcome in this sample was minimal and lacked practical significance. Figure 3 illustrates this relationship using the boxplots.

### Stigma as a predictor of successful TB treatment outcome

Overall, 76/87 (87%) of PWTB had successful TB outcomes. Bivariate logistic regression was used to assess the predictive ability of internalized and enacted stigma for overall TB treatment outcome. There was a significant association between internalized stigma and overall TB treatment outcome (OR 2.61; 95% CI 1.30, 5.23; *p* = 0.007) (Table 4). The study found that patients (*n* = 20) who reported experiencing low internalized stigma had 2.61 times more chance of having successful TB outcomes. For enacted stigma, the increased likelihood of patients (*n* = 18) who experienced low enacted stigma was also significantly associated with overall TB treatment outcome (OR 2.91; 95% CI 1.28, 6.62; *p* = 0.011) (Refer to Table 4). The study was able to show that patients with low levels of internalized stigma were 2.6 times more likely to have favorable TB treatment outcomes compared to patients with high internalized stigma. Similarly, patients with low levels of enacted stigma were 2.9 times more likely to have favorable TB treatment outcomes compared to patients with high enacted stigma.

**Table 4:** Bivariate logistic regression model between internalized stigma, enacted stigma and overall TB treatment outcome.

Covariate	Odds ratio	Std. err.	*z*	*P* > |*z*|	95% CI
Stigma (internal)					
Experienced some internal stigma	2.61	0.93	2.69	0.007	1.30–5.23
Stigma (enacted)					
Experienced some enacted stigma	2.91	1.22	2.54	0.011	1.28–6.62

The predictive ability of internalized stigma and enacted stigma scores on overall TB treatment outcome.

## Discussion

Tuberculosis stigma remains a barrier to early diagnosis and treatment completion (Mbuthia et al., 2020). In the context of on-completion of treatment for TB, patients’ failure to adhere to TB treatment is a major significant challenge that contributes to poor treatment outcomes (Gashu et al., 2021). Medication non-adherence is not only associated with poorer health outcomes but is one of the most significant obstacles to TB control globally (Kvarnström et al., 2021; Gebreweld et al., 2018). Furthermore, Subu et al. (2021) argue that stigma generally leads to negative social experiences, such as isolation, rejection, marginalization, and discrimination. Thus, if stigma is related to a health condition, it may affect a person’s illness and treatment course, including access to appropriate and professional medical treatment (Subu et al., 2021).

Given that stigma emanates from multiple levels outside the individual (i.e., family, community, institutions), understanding TB stigma is essential for improved interventions to minimize its effects (Mbuthia et al., 2020). The National Strategic Plan for HIV, TB, and sexually transmitted infections recognizes both HIV-related and TB-related self-stigma as major public health concerns. As a result, it aimed to halve both HIV-related and TB-related self-stigma by 2022 (National Department of Health, 2017). Evidence suggests that TB stigma in relation to treatment adherence and completion has been replete with contradictory results despite using prospective designs (Yan et al., 2018). A plausible explanation for these contradictions is the use of unvalidated scales to distinguish between and measure the different domains of stigma (Bresenham et al., 2020). Thus, additional quantitative work is warranted to assist stakeholders, such as TB program implementers or policymakers, in understanding the magnitude of stigma’s impact in specific settings or distinct subpopulations (Bresenham et al., 2020). The current findings fill this gap by using a validated stigma scale that measures both internalized and enacted stigma experienced by DS-TB patients.

The current findings supported that stigma (both internalized and enacted) predicted successful treatment outcomes. Patients with low levels of internalized and enacted stigma were 2.6 and 2.9 times (respectively) more likely to have successful TB treatment outcomes compared to patients with high internalized and enacted stigma. The impact of stigma is confirmed by several studies related to other chronic illnesses. In a scoping review of health-related stigma outcomes for high-burden diseases (like TB) in low and middle-income countries (Kane et al., 2019), stigma was associated with decreased medication adherence and, among patients with substance use, relapse. Another study conducted on TB and its determinants in Dalian, Northeast China, showed that stigma (i.e., experienced, anticipated and internalized) among TB patients was high (Chen et al., 2021). There is a growing awareness of the need to address the stigma related to TB (Craig et al., 2017), since stigma has long been recognized as a factor influencing medication adherence (Náfrádi et al., 2017). This could be because patients may not want others, or anybody, to know about their illness, owing to the fear of being stigmatized. The fear can be so intense that the patient would prefer not to take medication if there is a possibility that someone might be watching (Kvarnström et al., 2021).

The fear of TB transmission was also identified by Mbuthia et al. (2020) as a key driver of TB stigma among TB patients in a rural Kenyan community. Furthermore, TB stigma was represented through patients being isolated by others, self-isolation, fear of disclosing a TB diagnosis, association of TB with HIV, and lack of social support (Bresenham et al., 2020; Mbuthia et al., 2020). A study conducted by Abdisa et al. (2020) on self-stigma and medication adherence among patients with mental illness in South West Ethiopia found that self-stigma was a contributing factor to non-adherence to medication. That non-adherence contributes to increased healthcare costs (Abdisa et al., 2020).

In the context of South Africa, TB stigma has been investigated alongside HIV stigma owing to the contextual double burden of disease (DeSanto et al., 2023; Wouters et al., 2020). Recent statistics from TB DIAH Project (2024) highlight low levels of TB treatment success in South Africa (78.97%), but this study reported a success rate of 86%. This may be due to the low stigma experienced by the study participants, which, in turn, would not only foster a positive attitude toward treatment but also promote treatment compliance. DeSanto et al. (2023) recommended community and individual education campaigns on TB treatment and transmission, as well as training for health care workers on stigma and stigmatization interventions to prevent discrimination and protect patient confidentiality.

TB remains a pressing health concern in South Africa. Therefore, understanding stigma and how it interacts with patient progression along the TB cascade provides insight into specific groups of patients, especially those at greater risk for poor TB outcomes. This study supports the notion of Pradhan et al. (2022) of the urgent need to integrate mental health services into TB control programs. Based on the study’s findings, it is recommended that interventions be implemented to minimize stigma and lower the likelihood of early treatment interruption. Bresenham et al. (2020) recommend education initiatives to raise awareness and knowledge of TB treatment, aimed at minimizing social stigma. It is also recommended that further studies be conducted to understand how TB stigma functions and influences community perceptions. This information will not only contribute to lessening TB stigmatization but also improve treatment and retention in care. The TB program, health professionals, and policymakers are urged to consider the study’s recommendations for overcoming self-stigma to promote medication adherence among patients with TB disease.

## Limitations

A limitation of this study is that the sample of 90 participants who were receiving TB treatment was a convenience sample and not representative of all DS-TB patients in the country. The findings can thus not be generalized. In addition, the study design did not use randomization and a control group, therefore limiting its ability to establish a cause-and-effect relationship and increasing the risk of various biases. The experiences of low stigma may have been influenced by the registered nurses who could have interfered by motivating participants to take their medication to get better. The SSCI-8, however, was reliable and showed high internal consistency.

## Conclusion

The study showed that low stigma is associated with favorable TB outcomes among newly diagnosed TB patients. The measurement of stigma among TB patients can thus predict successful treatment outcomes. Taking a proactive approach is key to identifying earlier stigma experienced by DS-TB patients. Those experiencing stigma can be supported through interventions aimed at stigma reduction, improved treatment, and retention in care, ultimately yielding successful treatment outcomes.

The findings of this study can thus be used to design effective interventions that promote stigma reduction to reduce the probability of early treatment interruption. In addition, these interventions will not only help reduce TB stigmatization but also improve treatment and retention in care. The study thus contributes to understanding stigma in PWTB for policy development and interventions aimed at improving TB treatment outcomes.

## Data Availability

The raw data supporting the conclusions of this article will be made available by the authors, without undue reservation.
